# Football Compared with Usual Care in Men with Prostate Cancer (FC Prostate Community Trial): A Pragmatic Multicentre Randomized Controlled Trial

**DOI:** 10.1007/s40279-018-1031-0

**Published:** 2018-12-01

**Authors:** Eik Dybboe Bjerre, Klaus Brasso, Anders Bojer Jørgensen, Thomas Hindborg Petersen, Alexandra Röthlin Eriksen, Anders Tolver, Jesper Frank Christensen, Mads Hvid Poulsen, Søren Sørensen Madsen, Peter Busch Østergren, Michael Borre, Peter Krustrup, Christoffer Johansen, Mikael Rørth, Julie Midtgaard

**Affiliations:** 1 The University Hospitals’ Centre for Health Research, Rigshospitalet, 2100 Copenhagen Ø, Denmark; 2 Department of Urology, Rigshospitalet, Copenhagen Prostate Cancer Center, University of Copenhagen, 2100 Copenhagen Ø, Denmark; 3 Data Science Laboratory, Department of Mathematical Sciences, University of Copenhagen, 2100 Copenhagen Ø, Denmark; 4 Centre of Inflammation and Metabolism/Centre for Physical Activity Research, Rigshospitalet, 2100 Copenhagen Ø, Denmark; 5 Department of Urology, Odense University Hospital, 5000 Odense C, Denmark; 6 Department of Urology, Hospital of Southwest Denmark Esbjerg, 6700 Esbjerg, Denmark; 7 Department of Urology, Herlev and Gentofte University Hospital, 2730 Herlev, Denmark; 8 Department of Urology, Aarhus University Hospital, 8200 Aarhus N, Denmark; 9 Department of Sports Science and Clinical Biomechanics, University of Southern Denmark, 5230 Odense M, Denmark; 10 Sport and Health Sciences, College of Life and Environmental Sciences, University of Exeter, Exeter, UK; 11 Unit of Survivorship, Danish Cancer Society Research Center, 2100 Copenhagen Ø, Denmark; 12 Department of Oncology, Rigshospitalet, University of Copenhagen, 2100 Copenhagen Ø, Denmark; 13 Department of Public Health, University of Copenhagen, 2100 Copenhagen Ø, Denmark; 14 Academy of Geriatric Cancer Research (AgeCare), Odense University Hospital, 5000 Odense C, Denmark; 15 Finsen Center, Rigshospitalet 9601, 2100 Copenhagen, Denmark

## Abstract

**Background:**

Physical activity has been shown to mitigate the unwanted psychological and physiological side effects of prostate cancer treatments, but sustainable exercise possibilities are limited.

**Objective:**

Our objective was to examine whether football in a real-world setting (i.e., local football clubs) was safe and feasible in practice and could improve quality of life, mitigate decline in muscle mass and bone density, and increase fat mass in patients with prostate cancer.

**Methods:**

In this pragmatic, multicentre, parallel randomized controlled trial, men diagnosed with prostate cancer were recruited from five Danish urological departments. Men (*N* = 214) diagnosed with prostate cancer were randomly allocated, using random generated lists (block size 4–8) stratified for center and androgen-deprivation therapy status, to either 1 h of football twice weekly in a local football club or to usual care, which was a 15- to 30-min telephone session covering their options for physical activity or free-of-charge rehabilitation delivered as standard in Denmark. Allocation was concealed from the trial investigator performing the randomization, but—given the nature of the intervention—this was not possible for personnel and participants. Assessments were performed at baseline, 12 weeks, and 6 months. The primary outcome was mean change difference in prostate cancer-specific quality of life at 12 weeks. Secondary outcomes were body composition, 12-Item Short Form Health Survey (SF-12) physical and mental health, and safety—reported as fractures, falls, and serious adverse events.

**Results:**

Attrition was 1 and 3% at 12 weeks, and 5% and 5% at 6 months for the usual care and football groups, respectively. Prostate cancer-specific quality of life was equal between groups at 12 weeks (mean difference + 1.9 points, 95% confidence interval [CI] –1.0–4.8; *P* = 0.20) and at 6 months (+ 0.5 points, 95% CI –2.8–3.8; *P* = 0.76). Fractures were equally distributed, with two fractures in the usual care group and one in the football group. Likewise, body composition outcomes were equal. Mental health improved after 6 months of football (mean difference + 2.7 points, 95% CI 0.8–4.6; *P* = 0.006).

**Conclusions:**

In this trial, community-based football was a feasible exercise strategy for men with prostate cancer. Football did not improve prostate cancer-specific quality of life but did improve mental health; the clinical significance of this is unclear.

**Trial registration:**

ClinicalTrials.gov: NCT02430792.

**Electronic supplementary material:**

The online version of this article (10.1007/s40279-018-1031-0) contains supplementary material, which is available to authorized users.

## Key Points

**Table:** 

Systematic reviews of efficacy trials have shown that exercise is efficacious in improving quality of life, fatigue and exercise capacity. The current evidence base is limited regarding the evaluation of effectiveness of exercise strategies that can be sustained in the long term.
The results of this trial suggest that football implemented in local clubs for men with prostate cancer is a feasible exercise strategy and yields results comparable to those from being referred to physical activity and rehabilitation.
Football was low cost, and a majority of the men continued in the football club after the study period.

## Background

With 5.6 million men living with prostate cancer globally, and 1.4 million new diagnoses annually, the disease is not only the most frequent cancer among men but also a leading cause of preterm mortality in high- to middle-income countries [1, 2]. Treatment options vary according to patient and cancer characteristics, and most treatments induce unwanted psychological and/or physiological side effects [3]. In the case of localized disease, patients are offered curative treatment, including surgery. However, when disease is recurrent and/or advanced at the time of diagnosis, patients are offered androgen-deprivation therapy (ADT), with an estimated one in two patients receiving ADT at one point during their treatment course [4]. ADT has unintended effects, such as weight gain due to increase in fat mass, decrease in bone and lean muscle mass as well as development of metabolic syndrome and loss of masculinity [3, 5–7]. Some of these may be preventable or reversible with exercise [8, 9]. In addition, physical activity has been shown to reduce both overall and prostate cancer-specific mortality by 33% and 35%, respectively, independent of whether patients received ADT [10]. Trials testing the efficacy of exercise for patients with prostate cancer have demonstrated positive results [8, 9], but supporting long-term adherence and incorporating exercise into already existing infrastructures is challenging [11]. No trials fulfilling pragmatic design criteria have previously been identified in the field of exercise in men with prostate cancer [9]. Pragmatic trials are essential to close the gap between practice and science, as they are conducted in a real-world setting, include outcome measures that are relevant to patients, and are targeted at reducing resource use to support subsequent implementation and dissemination of the intervention [12]. We previously conducted a small-scale explanatory randomized controlled trial exploring the physiological effects of football training and demonstrated improved muscle strength and greater lean body mass and hip bone mineral density [13, 14]. These promising findings prompted us to examine the real-world effectiveness of this intervention when delivered in pre-existing infrastructure (i.e., local football clubs) [15]. Sports participation in a real-world setting is often referred to as a usable public health strategy for promoting physical activity [16, 17] but has never been assessed, to our knowledge, in a pragmatic randomized controlled trial that includes a clinical population.

We therefore designed the Football Club (FC) Prostate Community trial to examine the effectiveness and safety of community-based football compared with usual care. We hypothesized that football would be effective in improving quality of life (QoL), mitigating decline in muscle mass and bone density, and increasing fat mass.

## Methods

### Trial Design

The FC Prostate Community trial was a pragmatic, multicentre, randomized controlled trial (1:1) comparing community-based football with standard (usual) care. The trial was performed in accordance with the Helsinki Declaration and Good Clinical Practice (GCP) principles. The Ethics Committee for the Capital Region of Denmark (H-2-2014-099) and the Danish Data Protection Agency approved all of the centers involved. No changes were made during the conduct of the trial in relation to the original protocol [18] or trial registration.

### Setting and Participants

Eligible participants were men diagnosed with prostate cancer, able to complete trial documents in Danish, and willing to sign informed consent. Participants were excluded if their treating physician proscribed participation in football or if they had a *T* score below the criterion for osteoporosis (i.e., *T* score < 2.5 for spine or hip). Patients undergoing prostatectomy were not enrolled until 6 weeks after surgery.

The first patient was enrolled in June 2015 and the last in February 2017. Five Danish urological departments recruited patients. Patients were recruited by referral when attending follow-up appointments at their local outpatient urology clinic. Recruitment material was also available at local community centers that provide usual care rehabilitation.

### Randomization and Masking

Patients were randomly allocated to either a football group (FG) or a usual care group (UG) according to a computer-generated list of random numbers after all baseline assessments were completed. A statistician not otherwise involved in the trial generated separate lists with a 1:1 ratio and varying block sizes (*n* = 4–8) stratified for each center and treatment category (receiving ADT or not) using SAS. The allocation was concealed from trial personnel as the statistician received a password-protected email from the trial management system (EasyTrial^®^) with an upload function for the allocation sequence. After participants were enrolled by trial personnel at each hospital, the trial manager telephoned participants who had provided written informed consent and undergone all baseline measurements. Randomization was done using the web-based trial management system, and the participants were told which group they were allocated to. Given the nature of the intervention, neither participants nor coaches were blinded. Blinding was implemented for objective outcomes, so personnel performing the dual-energy X-ray absorptiometry (DXA) assessments had no information on the group allocation.

### Interventions

A detailed description of the intervention and procedures has been published in the trial protocol [18], and the development of the educational programme for the football coaches has been described in a qualitative study [19]. All participants were informed during a telephone session, in which they were also randomized, of the guidelines for physical activity for cancer survivors: to be physically active > 30 min daily and > 10 min of vigorous activity twice a week [20].

#### Football Group

FG participants were invited to 6 months of recreational football for 1 h twice weekly at a local football club. A start-up day was decided, and participants were given the local football coach’s contact information and told that all communication on adherence, injuries, and other football matters was to be handled by their coach. After the 6-month period, participants had the opportunity to continue the intervention by joining the football club on the local club’s terms, including paying the membership fee. Football sessions were scheduled to last 1 h and comprised 20 min of warm-up based on the Fédération Internationale de Football Association (FIFA) 11 + program [21] with modified upper-body exercises and 20 min each of drills and match play. The football coaches were recruited by the local club and underwent an 8- to 10-h educational program on prostate cancer treatment, patient symptoms, football-specific functional tests, and the intervention manual. The intervention was developed to work in everyday life, as this is the natural setting for sports participation. The rationale also included aligning the intervention with traditional masculine ideals because the participants can be at risk of a loss of masculinity [5].

#### Usual Care Group

UG participants were told that they would not be offered football and received a 15- to 30-min telephone session covering their options for physical activity and free-of-charge rehabilitation delivered by the municipalities, which is standard practice in Denmark; the rehabilitation services offered varies across municipalities [22]. They subsequently received an email containing the same information.

### Outcomes

The primary outcome was mean change in total prostate cancer-specific QoL measured with the Functional Assessment of Cancer Therapy-Prostate (FACT-P) questionnaire [23] (Likert scoring 0–4; range 0–156; the highest score indicates the best QoL) assessed 12 weeks after randomization. The primary time point was chosen as 12 weeks post baseline, based on the assumption that placing the primary assessment of change in QoL earlier (i.e., 3 months post baseline) rather than later (i.e., 6 months post baseline) would limit the risk of response shift [24].

Objectively measured secondary outcomes assessed by DXA were mean change in lean body mass, fat mass, and hip, femoral neck, spine, and whole-body bone mineral density after 6 months (for information on scanners at each center, see the Electronic Supplementary Material [ESM]). Self-reported outcomes were physical activity measured with the short International Physical Activity Questionnaire (IPAQ) and general physical and mental health measured with the 12-Item Short Form Health Survey (SF-12). All participants, irrespective of allocation group, were followed-up using the same procedures. Safety outcomes were fractures and falls requiring medical assessment and any serious adverse events (SAEs) defined according to GCP [25]. To enable unbiased assessment between groups, the two safety outcomes were evaluated using a self-completed case report form administered via the web-based data capture system. The cost of delivering the program was calculated using the actual price paid to the football clubs to cover expenses for purchasing equipment and reimbursement of coaches. We report the fraction of FG participants who continued to play football after 6 months; continuers were defined as attending more than five training sessions in the period from 6 months to 1 year. The coaches used a tablet-based app to submit data on adherence to the intervention, injuries, and fidelity in delivering the intervention after each session.

### Statistical Analysis

#### Sample Size

The sample size calculation was based on detection of a minimal clinically important difference (MCID) of 6 points on the FACT-P questionnaire [26] at 12 weeks. Based on results from a pivotal exercise trial for men with prostate cancer [27], which reported a standard deviation (SD) of 10 FACT-P points, an SD of 15 FACT-P points was chosen for the calculation as we expected the participants to be more heterogeneous than those in the study by Segal et al. [27]. With a two-sided significance level of 5% and a power of 80%, we required a minimum of 100 participants in each group to detect an MCID.

#### Analysis of Primary and Secondary Outcomes

The main analyses of the primary, secondary, and safety outcomes were performed according to the intention-to-treat (ITT) principle. We assessed distributions of continuous variables for normality, and residuals were inspected to validate the models used.

If any single item was missing in FACT-P, the official scoring guidelines were applied, removing this item by multiplying the sum of the subscale with the number of the items in the subscale and then dividing by the number of items answered. No imputations of data from missing participants were performed as missing data occurred in < 3% of cases for the primary outcome and < 6% for any other outcomes. In the analysis of the primary outcome, we compared change score of disease-specific QoL score (total score of FACT-P) at 12 weeks between allocation groups using analysis of covariance, where we included the allocation group and adjusted for baseline value, age, and the stratification factor ADT status in the model. Changes are presented as marginal mean differences between allocation groups with 95% confidence intervals and p-values. For the secondary outcomes (lean body mass, fat mass, whole-body and regional bone measures) with two timepoints, we used the same approach as for the primary outcome. All figures delineate results from unadjusted models.

We used Fischer’s exact test to compare differences in proportions of the safety outcomes (fractures, falls, and SAEs).

We performed subgroup analyses defined a priori on participants undergoing ADT. We also performed post hoc subgroup analyses stratifying participants according to physical activity categories proposed in the official IPAQ scoring protocol [28]. These analyses were performed as described for the ITT population. To determine the effect of playing football, and not just being assigned to football, we performed per-protocol analyses on a priori defined criteria of 50% adherence. To address any confounding when comparing all UG participants with a subgroup that cannot be stratified for, we adjusted for lifestyle, demographics, and disease prognostic risk factors known at baseline as suggested by Hernán et al. [29]. For the same reason, we used a logistic regression incorporating the same risk factor variable to analyze the comparative safety outcomes of fractures, falls, and admissions.

Post hoc sensitivity analyses that included disease stage, treatment, and Gleason score were performed to examine whether heterogeneity influenced effect estimates. All analyses were conducted with STATA 15, version 1. The plan for the statistical analyses was published before any outcome data were collected on the trials registry site.

### Patient and Public Involvement

Two patient partners were involved in the trial’s steering group and contributed actively in all stages of the trial, including seeking funding, developing research questions, trial conduct, and discussing trial results and the manuscript draft. Specifically, patient partners in the steering group helped guide strategies to recruit patients, which also included help/advice from the national prostate cancer patient organization, which agreed to tell its members about the trial and to provide details about participation through newsletters.

The intervention manual was developed based on expert interviews and focus groups with patients and other stakeholders, e.g., volunteer football coaches and urology nurses, as described elsewhere [19].

Published reports of trial results will be emailed to participants and disseminated to patient advocates through the national prostate cancer organization and relevant scientific meetings and conferences.

## Results

### Study Sample

**Fig. 1:**
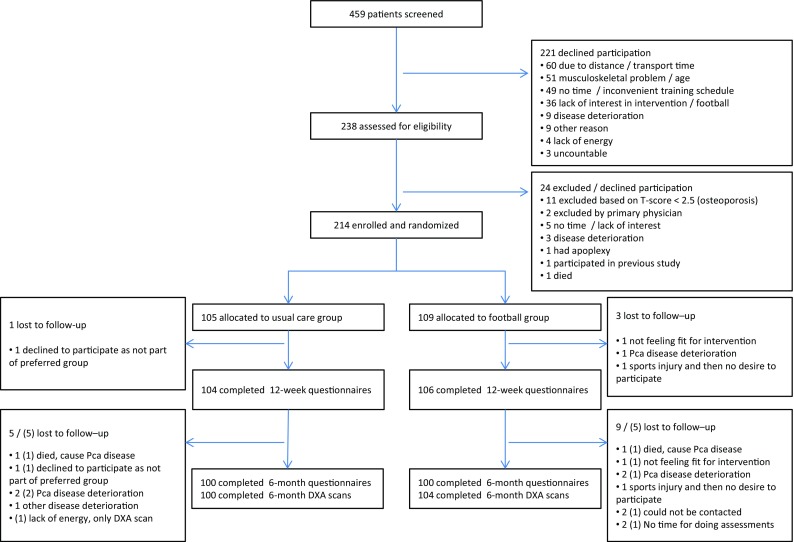
Flow of participants. *DXA* Dual-energy X-ray absorptiometry, *Pca* prostate cancer, *()* DXA lost to follow up

Between 15 June 2015 and 28 February 2017, we screened 459 patients, 238 (51%) of whom were assessed for eligibility, and 214 (90%) were randomized (Fig. 1). Geographic distance/travel time and poor physical condition were the two most prevailing reasons for declining participation in the trial. Of the 459 patients, 37 (8%) declined to participate, explicitly stating they had no interest in football. Table 1 presents the demographics and clinical characteristics (*n* = 214).

**Table 1:** Baseline characteristics of patients according to allocation group

Characteristic	FG (*n* = 109)	UG (*n* = 105)	Total (*n* = 214)
Age (years)	67.8 ± 6.2	69.0 ± 6.2	68.4 ± 6.2
Employment status			
Paid work	26 (24)	26 (25)	52 (24)
Unemployed	2 (2)	0 (0)	2 (1)
Sick leave	1 (1)	2 (2)	3 (1)
Retired	80 (73)	77 (73)	157 (73)
Education			
No education	7 (6)	5 (5)	12 (6)
Primary education (9th/10th grade)	4 (4)	5 (5)	9 (4)
Vocational education	33 (30)	28 (27)	61 (29)
Secondary education (12th grade)	10 (9)	15 (14)	25 (12)
Completed college or higher	55 (50)	52 (50)	107 (50)
Marital status			
Married or living with partner	92 (84)	93 (89)	185 (86)
Other (single, divorced, or widowed)	17 (16)	12 (11)	29 (14)
Smoking status			
Yes	17 (16)	11 (10)	28 (13)
No, stopped	49 (45)	51 (49)	100 (47)
No, never	43 (39)	43 (41)	86 (40)
Alcohol consumption (drinks per week)	9.1 ± 7.2	8.5 ± 7.0	8.8 ± 7.1
Time since diagnosis, years	3.0 ± 2.7	3.8 ± 3.9	3.4 ± 3.4
Risk group			
Localized, prostatectomized	16 (15)	15 (14)	31 (14)
Localized, not prostatectomized	27 (25)	28 (27)	55 (26)
Locally advanced	39 (36)	42 (40)	81 (38)
Metastatic	26 (24)	19 (18)	45 (21)
Unknown	1 (1)	1 (1)	2 (1)
ISUP Gleason Grading			
Group 1 (Gleason score 2–6)	15 (14)	13 (12)	28 (13)
Group 2 (Gleason score 3 + 4)	29 (27)	36 (34)	65 (30)
Group 3 (Gleason score 4 + 3)	18 (17)	13 (12)	31 (14)
Group 4 (Gleason score 8)	18 (17)	13 (12)	31 (14)
Group 5 (Gleason score 9–10)	28 (26)	24 (23)	52 (24)
Unknown	1 (1)	6 (6)	7 (3)
Number of men with bone metastasis	22 (20)	19 (18)	41 (19)
Current treatment at baseline			
No treatment^a^	46 (42)	42 (40)	88 (41)
Anti-androgen monotherapy	15 (14)	21 (20)	36 (17)
Castration (surgical or pharmacological)	46 (42)	41 (39)	87 (41)
Unknown	2 (2)	1 (1)	3 (1)
Previous treatment at baseline			
Prostatectomy	27 (25)	39 (37)	66 (31)
Radiation	37 (34)	29 (28)	66 (31)
ADT and radiation with curative intent	21 (19)	16 (15)	37 (17)
Chemotherapy (docetaxel)	9 (8)	10 (10)	19 (9)
No prior or current treatment	21 (20)	24 (22)	45 (21)
Time on ADT, median days (*n* = 88)	512.5 (208–881)	580 (235–1089)	520 (213–982)
Number of comorbidities			
Zero	35 (32)	29 (28)	64 (30)
One	45 (41)	46 (44)	91 (43)
Two	16 (15)	18 (17)	34 (16)
Three or more	13 (12)	12 (11)	25 (12)
Baseline values on outcomes			
Prostate cancer-specific QoL (FACT-P, points)	123.7 ± 17.3	124.6 ± 16.6	124.1 ± 16.9
Lean body mass (kg)	56.6 ± 6.3	57.5 ± 7.1	57.0 ± 6.7
Fat mass (kg)	27.5 ± 8.0	28.3 ± 8.9	27.9 ± 8.4
Whole-body BMC (kg)	3.1 ± 0.5	3.1 ± 0.6	3.1 ± 0.5
Whole-body BMD (g/cm^2^)	1.215 ± 0.116	1.219 ± 0.125	1.217 ± 0.120
Femoral neck BMD (g/cm^2^)	0.877 ± 0.155	0.877 ± 0.147	0.877 ± 0.151
Total hip BMD (g/cm^2^)	1.015 ± 0.132	1.025 ± 0.138	1.020 ± 0.134
Lumbar BMD (g/cm^2^)	1.188 ± 0.226	1.189 ± 0.223	1.188 ± 0.224
Weekly self-reported PA (median MET)^b^	3649 (1824–6693)	4098 (2394–7732)	4046 (2010–6845)

Data are presented as mean ± standard deviation, n (%) or median (interquartile range)

*ADT* androgen-deprivation therapy, *BMC* bone mineral content, *BMD* bone mineral density, *FACT*-*P* Functional Assessment of Cancer Therapy-Prostate questionnaire, *FG* football group, *ISUP* International Society of Urological Pathology, *MET* metabolic equivalent task, *PA* physical activity, *QoL* quality of life, *UG* usual care group

^a^Watchful waiting, active surveillance, previous prostatectomy, or radiation

^b^102 patients in the FG and 96 patients in the UG

In total, 105 patients were randomly allocated to the UG and 109 to the FG. Retention in the study was 98% at 12 weeks and 95% at 6 months. For the FG, median attendance from baseline to 12 weeks was 16 [interquartile ratio (IQR) 10] trainings (64%), and from baseline to 6 months it was 28 (IQR 19) trainings (59%). From baseline to 12 weeks, and from baseline to 6 months, 82 (75%) and 71 (65%) participants, respectively, had an attendance to the intervention of ≥ 50% defining the per-protocol population.

### Intervention

Fidelity to the intervention manual by football coaches was 3.8 (3.7–3.9) points on a 5-point Likert scale, where 1 was non-adherence and 5 was perfect adherence to the intervention manual. During the trial, 616 football sessions were conducted. On average, sessions lasted 58.8 (58.4–59.3) minutes, with 20.4 (20.0–20.8) minutes spent on warm-up, 16.1 (15.5–16.6) minutes on drills, and 22.3 (21.8–22.9) on match play.

### Outcomes

**Fig. 2:**
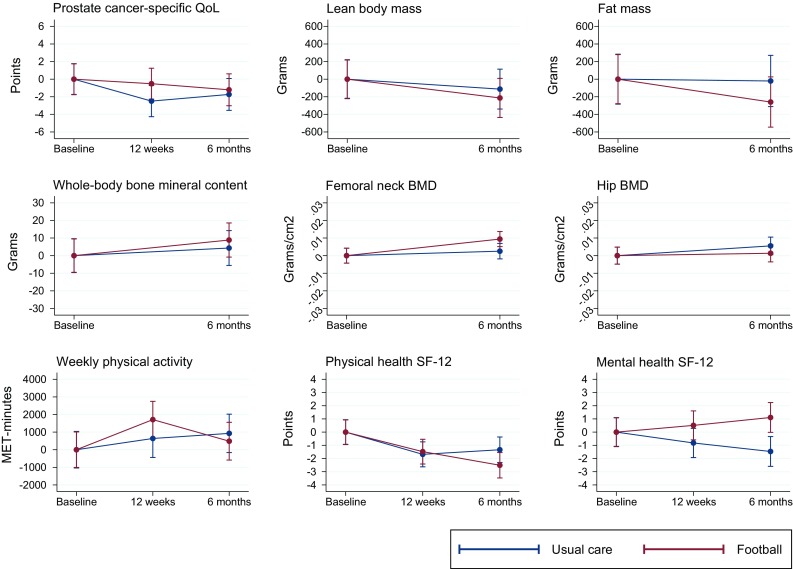
Mean change in outcomes, according to allocation group and time points. *QoL* quality of life, *BMD* bone mineral density

At 12 weeks, adjusted mean change on the FACT-P score was 1.9 points (95% CI − 1.0–4.8; *P* = 0.20) higher in the FG than in the UG. The FACT-P adjusted mean change value at 6 months was 0.5 points (95% CI − 2.8–3.8; *P* = 0.76) (Fig. 2).

**Fig. 3:**
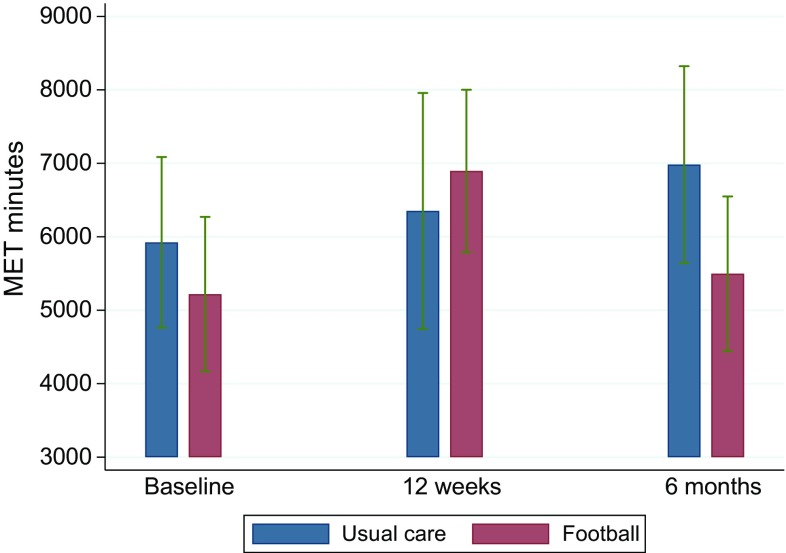
Weekly MET minutes, according to allocation group. *MET* metabolic Equivalent

At both 12 weeks and 6 months, the level of self-reported physical activity was similar between the FG and the UG (*P* = 0.46 and 0.34) (Table 2). A significant within-group increase in the FG was seen from baseline to 12 weeks, with an increase of 115 min in the vigorous activity category. This increase was completely reversed at 6 months (see Fig. 3).

**Table 2:** Outcomes for the intention-to-treat population according to group allocation

	FG (*n* = 109)		UG (*n* = 105)		Covariance analysis, difference between groups		Unadjusted analyses, *t* test, difference between groups	
	*N*	Mean (95% CI)	*N*	Mean (95% CI)	Adjusted for ADT, age, and baseline score	*P* value	Unadjusted	*P* value
Change in prostate cancer-specific quality of life (points; higher is better)								
12 weeks	106	− 0.5 (− 2.6 to 1.5)	104	− 2.5 (− 4.6 to − 0.4)	1.9 (− 1.0 to 4.8)	0.20	2.0 (− 0.9 to 4.9)	0.18
6 months	100	− 1.2 (− 3.6 to 1.2)	100	− 1.7 (− 4.1 to 0.6)	0.5 (− 2.8 to 3.8)	0.76	0.5 (− 2.8 to 3.9)	0.76
Change in lean body mass (kg)								
6 months	104	− 0.2 (− 0.5 to 0.1)	100	− 0.1 (− 0.4 to 0.2)	− 0.1 (− 0.6 to 0.3)	0.60	− 0.1 (− 0.6 to 0.4)	0.66
Change in fat mass (kg)								
6 months	104	− 0.3 (− 0.7 to 0.2)	100	0.0 (− 0.4 to 0.4)	− 0.3 (− 0.9 to 0.3)	0.28	− 0.2 (− 0.8 to 0.3)	0.42
Change in whole-body bone mineral content (g)								
6 months	104	8.9 (− 5.1 to 22.8)	100	4.3 (− 9.9 to 18.5)	3.2 (− 16.9 to 23.3)	0.75	4.5 (− 15.3 to 24.4)	0.65
Change in whole-body BMD (g/cm^2^)								
6 months	104	0.002 (− 0.002 to 0.007)	100	0.001 (− 0.003 to 0.006)	0.001 (− 0.006 to 0.007)	0.87	0.001 (− 0.006 to 0.007)	0.75
Femoral neck BMD (g/cm^2^)								
6 months	104	0.009 (0.003 to 0.016)	100	0.003 (− 0.003 to 0.009)	0.007 (− 0.002 to 0.016)	0.12	0.007 (− 0.002 to 0.016)	0.13
Change in total hip BMD (g/cm^2^)								
6 months	104	0.001 (− 0.006 to 0.008)	100	0.006 (− 0.002 to 0.013)	− 0.005 (− 0.015 to 0.005)	0.34	− 0.004 (− 0.014 to 0.006)	0.41
Change in lumbar spine L1–L4 BMD (g/cm^2^)								
6 months	104	0.014 (0.003 to 0.025)	99	0.011 (0.000 to 0.022)	0.003 (− 0.013 to 0.019)	0.72	0.003 (− 0.013 to 0.018)	0.74
Change in weekly physical activity (MET minutes)								
12 weeks	96	1710 (328 to 3092)	89	638 (− 797 to 2073)	698 (1158 to 2555)	0.46	1072 (− 920 to 3064)	0.29
6 months	90	485 (− 759 to 1729)	87	928 (− 337 to 2193)	− 754 (− 2316 to 808)	0.34	− 443 (− 2217 to 1332)	0.62
Change in general physical health (SF-12)								
12 weeks	106	− 1.5 (− 2.6 to − 0.4)	104	− 1.7 (− 2.8 to − 0.6)	0.1 (− 1.4 to 1.6)	0.92	0.2 (− 1.3 to 1.8)	0.79
6 months	100	− 2.5 (− 3.8 to − 1.2)	100	− 1.3 (− 2.6 to − 0.1)	− 1.1 (− 2.9 to 0.6)	0.21	− 1.2 (− 3.0 to 0.6)	0.20
Change in general mental health (SF-12)								
12 weeks	106	0.5 (− 0.8 to 1.8)	104	− 0.8 (− 2.1 to 0.5)	1.5 (− 0.2 to 3.3)	0.08	1.3 (− 0.5 to 3.2)	0.15
6 months	100	1.1 (− 0.4 to 2.6)	100	− 1.5 (− 2.9 to 0.0)	2.7 (0.8 to 4.6)	0.01	2.6 (0.5 to 4.7)	0.02

Data are presented as n or mean (95% confidence interval) unless otherwise indicated

*ADT* androgen-deprivation therapy, *BMD* bone mineral density, *CI* confidence interval, *FG* football group, *MET* metabolic equivalent, *SF*-*12* 12-Item Short Form Health Survey, *UG* usual care group

No significant differences in change between the two groups were seen in lean body mass, fat mass, and hip, femoral neck, spine, and whole-body bone mineral density (Table 2). For subgroup analyses prespecified for the ADT population, no difference from the ITT population was observed (see Table 2 in the ESM). Per-protocol analyses likewise yielded no difference from the results of the ITT population (see Table 4 in the ESM). To examine whether heterogeneity of participants affected results, sensitivity analyses were conducted that included disease stage, treatment, and Gleason score, but they did not change the results, e.g., the adjusted mean change on 12-week FACT-P score was 2.3 points (95% CI − 0.6–5.1; *P* = 0.12) higher in the FG than in the UG.

**Fig. 4:**
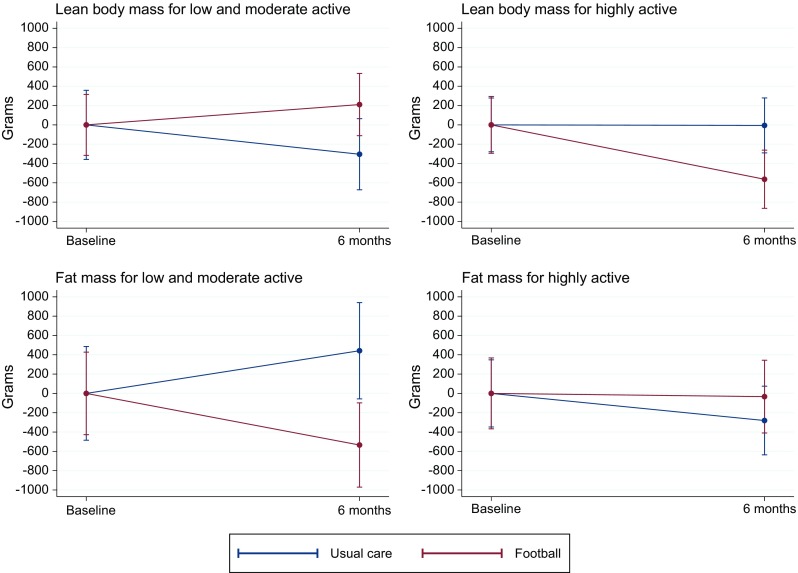
Mean changes in body composition based on baseline physical activity

In analyses where participants were stratified according to baseline physical activity level, participants who were categorised as being low or moderate physically active had a non-significant increase in lean body mass and a significant decrease in fat mass of 0.5 kg (95% CI − 0.9 to − 0.0; *P* = 0.04). The opposite pattern was seen for highly active participants, i.e., non-significant decrease in lean body mass and non-significant increase in fat mass (see Fig. 4).

For physical health (SF-12) at 12 weeks, the adjusted mean change was 0.1 points (95% CI − 1.4–1.6; *P* = 0.92) higher in the FG than in the UG. The score at 6 months was 1.1 points (95% CI − 2.9–0.6; *P* = 0.21) lower in the FG than in the UG. For mental health (SF-12) at 12 weeks, the adjusted mean change was 1.5 points (95% CI − 0.2–3.3; *P* = 0.08) higher in the FG than in the UG. The score at 6 months was 2.7 points (95% CI 0.8–4.6; *P* = 0.006) higher in the FG than in the UG.

### Safety Outcomes, Adverse Events, and Sports Injuries

FG participants reported ten falls compared with six falls in the UG (*P* = 0.44). Two fractures were reported in the UG and one in the FG (*P* = 1.00). Overall, 33 hospital admissions (SAEs by GCP definitions) were reported, 22 in the UG and 11 in the FG (*P* = 0.12) (see Table 1 in the ESM). One SAE related to the intervention was reported in the study and occurred due to an excoriation/scratch sustained when a shin guard scratched the lower leg. This later became infected and was not treated with the adequate antibiotic, resulting in a skin transplant from the thigh to the shin. The participant returned to football play after 8 weeks. Two deaths, one in each group and both due to prostate cancer, were reported. A total of 60 sports injuries (58 minor and two major) resulted in either ending a football session early or missing one or more football session. The most frequent injuries were muscle strain or sprains (*n* = 40), and the two major injuries were a partially and a fully ruptured Achilles tendon (see Table 6 in the ESM).

### Cost and Continuation

The cost of delivering the FG intervention was Danish Krone (DKK) 297,500 ($US46,213 on 17 October 2018). After the 6-month study period, 64 (59%) of the 109 participants allocated to football continued the intervention and joined their local football club.

## Discussion

This trial demonstrated it was possible to recruit and retain men with prostate cancer, with and without metastases and comorbidities, to community-based football. We found no differences in changes in prostate cancer-specific QoL or in body composition between the FG and the UG. However, at the 6-month follow-up, we found improvement in the exploratory outcome concerning the mental health domain of the SF-12 in the FG compared with the UG. The comparative safety outcomes demonstrated no differences in incidence rates of fractures, falls, and hospital admissions. FG participants experienced a substantial number of mostly minor sports injuries during the intervention. While these injuries did not lead to an increase in withdrawal from/termination of the intervention, they may have contributed to the decrease in physical activity after 12 weeks in participants recovering from their sports injury. A concurrent qualitative study including FG participants who had sustained an injury indicated that participants viewed injuries as a largely acceptable, intrinsic feature of football that also provided an opportunity to embody masculine ideals (unpublished data). We had expected a general deterioration in QoL and body composition outcomes in the UG because of the natural course of the disease and the side effects of treatments [6, 30, 31]. However, in light of the self-reported physical activity data, which delineated that both groups were highly physically active, the finding on equality is not surprising. Physical activity reported at baseline also shows that our sample of patients with prostate cancer was highly physically active before randomization. Therefore, we performed sensitivity analyses separating highly physically active participants from those who were only low or moderately active. As Fig. 4 shows, different patterns were observed for changes in muscle mass and fat mass in the two populations, indicating that football might only benefit patients who are less physically active. This is in line with other findings in a recently reported exercise trial for men with prostate cancer [32].

A previous study by Uth et al. [13] that was optimized to explore the causal effects of football, i.e., comparing football (delivered in an ideal setting) with no football, showed an improvement in lean body mass of 700 g favoring football. The hypothesis for the current pragmatic trial was that football would be a more potent intervention than the standard usual care (rehabilitation offered by local authorities), but the present findings did not confirm this. The discrepancy between the findings in the two trials might be due to differences in the interventions in the comparison groups. It can be argued that control group participants perhaps are less likely to find physical activity interventions by themselves knowing that they will be offered an intervention after 12 weeks, which was the case in the previous explanatory trial. In the current pragmatic trial, control group participants knew they would not be offered the football intervention for a whole year. With regard to attendance and workload during football training, our data show results similar to those from the previous explanatory trial [33]. In the explanatory trial, where participants were coached by a professional sport physiologist, participants ran 905 ± SD 297 m at speeds > 6 km/h and 2646 ± 705 m per session. In comparison, activity profiles sampled conveniently (*n* = 67) in the current pragmatic trial with local coaches showed that participants ran 973 ± 582 at running speeds > 6 km/h and a total of 2684 ± 918 m per session.

Several recently published systematic reviews on exercise for men with prostate cancer have outlined that exercise improves QoL, fatigue, fitness, and functional outcomes [8, 9, 34–36]. The studies generally focused on delivering exercise in healthcare clinics. Recent studies have focused on safety, feasibility, and initial efficacy of physical exercise for patients with prostate cancer with skeletal metastases [37, 38]. Even though the current trial was not designed to explore the issue of safety for this subpopulation, we enrolled 41 patients with skeletal metastases. We tested whether men with metastases had fewer training sessions than those without metastases and found this not to be the case (data not shown). This suggests that future studies powered and designed to test whether vigorous exercise is safe for men with bone metastases are possible.

We conducted a qualitative study, which indicated that men with prostate cancer undergoing ADT viewed football as an opportunity to take back control and responsibility for their own health without being in the role of patient, and that football enabled peer-to-peer caring behavior in a male setting [39]. This can be interpreted as being in alignment with the improvement in mental health, which at the same time should be interpreted with caution, as this was an exploratory outcome chosen primarily for the purpose of conducting health economic analysis. Only 4% (data not shown) of participants played football before entering the study and 57% joined their local club and continued play after the study period.

The financial cost of delivering the football intervention was DKK2600 ($US404) per participant. In comparison, a report by the Danish National Centre for Social Research estimated the average cost of rehabilitation delivered by two different local authorities in Denmark to be DKK29,211 ($US4538) for 12.9 weeks of rehabilitation and DKK32,478 ($US5045) for 10.8 weeks per patient [40].

Collectively, this information supports the suggestion that sports participation is appealing and feasible as a public health strategy for clinical populations [16, 17].

The trial had various methodological strengths. First, assessments and DXA scans were performed by blinded assessors, and the self-reported questionnaires were automated via a web-based system, helping to minimize errors and limit the influence of trial staff on participants. Safety outcomes and SAEs were followed-up using the same method for all participants; at the same time, all injuries related to the intervention were collected prospectively. Second, attrition bias was minimal as retention was 98 and 95%. Third, the trial followed GCP principles, ensuring homogeneity between study sites.

Limitations include the obvious inability to blind participants and coaches delivering the intervention. While this can induce bias on the self-reported outcomes, these measures yielded similar results to the objectively measured and assessor-blinded outcomes. When claiming equality between groups, several methodological features need to be considered: the sample size should be large because CIs should not exceed a certain marginal size, which preferably should have been set in advance, though this was not the case in this trial. In the interpretation of the trial results, we used the delta values stated a priori from our original sample size calculations, i.e., the MCID of 6 points for FACT-P [26] and a 700 g change in lean body mass from Uth et al. [13]. We found that 95% CIs did not exceed these margins. We observed lesser variations than expected in outcomes, which is why the trial had more power than anticipated. Last, a trial can wrongfully claim equivalence in safety if a sufficient proportion of participants are lost to follow-up or if participants not receiving the intervention are diluting the estimates of the ITT analysis. This trial had a negligible withdrawal rate, and we performed per-protocol analysis as sensitivity analyses to address any dilution (see the ESM). In regard to the per-protocol population defined as adherence of 50%, a limitation is that only two sessions were scheduled per week and higher attendance could have incurred possible benefits, especially for the physiological outcomes. Regarding generalizability, the pragmatic multicentre design is valuable as it ensures that the estimated results reflect a real-world implementation of community-based football compared with relevant alternatives. One issue concerning the pragmatic design is whether the heterogeneity of participants potentially reduced the ability to infer validly on the measured outcomes. For this reason, we performed post hoc sensitivity analyses with any baseline covariate that might be a prognostic factor to test whether this would change the results. However, it only changed the results minimally and did not lead to any difference in interpretations. We also report subgroup analyses for the ADT population, which, as the width of CIs shows, was adequately powered with a total of 87 participants.

## Conclusion

In the current trial, football implemented in local football clubs under the supervision of lay coaches was a feasible exercise strategy for patients with prostate cancer. It improved the exploratory outcome mental health but offered no additional benefits with respect to prostate cancer-specific QoL or body composition after 6 months compared with encouragement/referral to physical activity and rehabilitation. As the risk of sports injuries seems to be an embedded aspect of participating in football, patients wishing to partake must accept this. Future research on sport, exercise, and physical activity should be conducted in real-world structures to enable low-cost upscaling.

## Electronic supplementary material

Below is the link to the electronic supplementary material.

Supplementary material 1 (PDF 741 kb)

## Abbreviations

ADT: Androgen deprivation therapy
GCP: Good Clinical Practice
FG: Football Group
UG: Usual care Group
DXA: Dual-energy X-ray Absorptiometry
QoL: Quality of Life
FACT-P: Functional Assessment of Cancer Therapy-Prostate
IPAQ: International Physical Activity Questionnaire
SF-12: 12-Item Short Form Health Survey
SAEs: Serious Adverse Events
MCID: Minimal clinical important difference
SD: Standard Deviation
ITT: Intention-to-treat
IQR: Interquartile range
CI: Confidence Interval
