# Supplementary material for: Football Compared with Usual Care in Men with Prostate Cancer (FC Prostate Community Trial): A Pragmatic Multicentre Randomized Controlled Trial
Source: Sports Med. 2018 Dec 1;49(1):145–58. doi: 10.1007/s40279-018-1031-0 (PMC6349963; doi:10.1007/s40279-018-1031-0)
Supplement: Supplementary file 1 — Supplementary material 1 (PDF 741 kb) [file 40279_2018_1031_MOESM1_ESM.pdf]

Electronic Supplementary Material

This supplementary material has been provided by the authors to give readers additional information about their work.

Supplement to: Bjerre ED[1], Brasso K[2], Jørgensen AB, BSc[3], Petersen TH[3],Eriksen AR[3], Tolver A[4], Christensen JF[5], Poulsen MH[6], Madsen SS[7], Østergren PB[8], Borre M [9],Krustrup P[10], Johansen C [11], Rørth M [12], Midtgaard J[13]. Football compared to usual care in men with prostate cancer (FC Prostate Community trial): a pragmatic multicentre randomised controlled trial. Sports Medicine.

Corresponding author: Eik Dybboe Bjerre; eb@ucsf.dk, telephone: +45 22749076 ORCID ID: 0000-0001-9370-5162

Affiliations:

1) The University Hospitals' Centre for Health Research, Rigshospitalet, 2100 Copenhagen Ø, Denmark
2) Copenhagen Prostate Cancer Center, Department of Urology, Rigshospitalet, University of Copenhagen, 2100 Copenhagen Ø, Denmark
3) The University Hospitals' Centre for Health Research, Rigshospitalet, 2100 Copenhagen Ø, Denmark
4) Data Science Laboratory, Department of Mathematical Sciences, University of Copenhagen, 2100 Copenhagen Ø, Denmark
5) Centre of Inflammation and Metabolism / Centre for Physical Activity Research, Rigshospitalet, 2100 Copenhagen Ø, Denmark;
6) Department of Urology, Odense University Hospital, 5000 Odense C, Denmark
7) Department of Urology, Hospital of Southwest Denmark Esbjerg, 6700 Esbjerg, Denmark
8) Department of Urology, Herlev and Gentofte University Hospital, 2730 Herlev, Denmark
9) Department of Urology, Aarhus University Hospital, 8200 Aarhus N, Denmark
10) University of Southern Denmark, Department of Sports Science and Clinical Biomechanics, 5000 Odense C, Denmark & Sport and Health Sciences, College of Life and Environmental Sciences, University of Exeter, United Kingdom
11) Danish Cancer Society Research Center, 2100 Copenhagen Ø, Denmark
12) Department of Oncology, Rigshospitalet, University of Copenhagen, 2100 Copenhagen Ø, Denmark
13) The University Hospitals' Centre for Health Research, Rigshospitalet, 2100 Copenhagen Ø, Denmark and Department of Public Health, University of Copenhagen, 2100 Copenhagen Ø, Denmark

ESM Table 1 Safety outcomes for ITT Population

	Football group (n=109)	Usual care group (n=105)
Safety outcomes		
Falls	10	6
p-value p=0.44		
Fractures	1	2
p-value p=1.00		
Serious adverse events (admissions)		
One	9	8
Two	1	7
Total	11	22
p-value p=0.12		

ESM Table 2 Outcomes for ADT population, according to group allocation

	Football group (n=46)		Usual care group (n=41)		Covariance analysis, difference between groups, mean (95% CI)		Unadjusted analyses, t-test, difference between groups, mean (95% CI)	
	n	Mean (95% CI)	n	Mean (95% CI)	Adjusted for ADT, age, and baseline score	p value	Unadjusted	p value
Change in prostate cancer-specific quality of life (points, higher is better)								
12 weeks	43	-1.3 (-4.6 to 1.9)	40	-3.5 (-6.8 to 0.2)	2.2 (-2.3 to 6.6)	0.33	2.1 (-2.5 to 6.7)	0.36
6 months	42	-3.5 (-6.8 to -0.3)	37	-4.6 (-8.0 to -1.1)	0.9 (-3.9 to 5.7)	0.71	1.0 (-3.7 to 5.8)	0.67
Change in lean body mass (kilograms)								
6 months	44	-0.2 (-0.8 to 0.3)	37	-0.5 (-1.0 to 0.1)	0.2 (-0.7 to 1.0)	0.69	0.2 (-0.6 to 1.0)	0.58
Change in fat mass (kilograms)								
6 months	44	-0.2 (-0.8 to 0.5)	37	0.2 (-0.5 to 0.9)	-0.4 (-1.4 to 0.6)	0.42	-0.4 (-1.4 to 0.5)	0.39
Change in whole-body bone mineral content (grams)								
6 months	44	22.8 (0.1 to 45.5)	37	0.2 (-22.0 to 27.0)	22.0 (-12.4 to 56.3)	0.21	20.6 (-13.0 to 54.2)	0.23
Change in whole-body bone mineral density (grams/cm^2)								
6 months	44	0.005 (-0.002 to 0.013)	37	-0.001 (-0.009 to 0.008)	0.005 (-0.007 to 0.017)	0.40	0.006 (-0.006 to 0.017)	0.31
Femoral neck BMD (grams/cm^2)								
6 months	44	0.009 (-0.002 to 0.019)	37	0.003 (-0.009 to 0.014)	0.007 (-0.009 to 0.023)	0.39	0.006 (-0.009 to 0.021)	0.47
Change in total hip BMD (grams/cm^2)								
6 months	44	0.005 (-0.011 to 0.021)	37	0.015 (-0.003 to 0.032)	-0.009 (0.033 to 0.014)	0.43	-0.010 (-0.033 to 0.014)	0.42
Change in lumbar spine L1-L4 BMD (grams/cm^2)								
6 months	44	0.025 (0.001 to 0.050)	37	0.013 (-0.014 to 0.039)	0.017 (-0.019 to 0.053)	0.34	0.013 (-0.023 to 0.048)	0.48
Change in weekly physical activity (MET-minutes)								
12 weeks	40	2405 (-396 to 5205)	32	3174 (43 to 6305)	-845 (-4920 to 3229)	0.68	-770 (-4970 to 3431)	0.72
6 months	38	1060 (-475 to 2595)	31	1505 (-195 to 3204)	-312 (-2501 ton 1878)	0.78	-444 (-2734 to 1845)	0.70
Change in general physical health (SF-12)								
12 weeks	43	-1.4 (-3.0 to 0.3)	40	-1.8 (-3.5 to -0.1)	-0.0 (-2.3 to 2.2)	0.98	0.4 (-1.9 to 2.8)	0.72
6 months	42	-2.7 (-4.9 to -0.4)	37	-1.8 (4.1 to 0.6)	-1.0 (-4.2 to 2.1)	0.51	-0.9 (-4.1 to 2.4)	0.59
Change in general mental health (SF-12)								
12 weeks	43	0.3 (-1.9 to 2.5)	40	-2.0 (4.2 to 0.3)	2.5 (-0.2 to 5.3)	0.07	2.3 (-0.8 to 5.4)	0.15
6 months	42	-0.2 (-2.8 to 2.4)	37	-3.7 (-6.5 to -1.0)	3.5 (-0.1 to 7.2)	0.06	3.5 (-0.3 to 7.3)	0.07

ESM Table 3 Safety outcomes for ADT Population

	Football group (n=46)	Usual care group (n=41)
Safety outcomes		
Falls	5	4
p-value p=1.00		
Fractures	1	1
p-value p=1.00		
Serious adverse events (admissions)		
One	4	1
Two	0	3
Total	4	7
p-value p=0.11		

ESM Table 4 Outcomes for per-protocol population, according to group allocation

(Minimum attendance for football 50%)	Football group		Usual care group		Covariance analysis, difference between groups, mean (95% CI)		Unadjusted analyses, t-test, difference between groups, mean (95% CI)	
	n	Mean (95% CI)	n	Mean (95% CI)	Adjusted for risk variables*	p value	Unadjusted	p value
Change in prostate cancer-specific quality of life (FACT-P total score, higher is better)								
12 weeks	82	-0.7 (-3.1 to 1.7)	104	-2.5 (-4.6 to -0.4)	3.0 (-0.2 to 6.1)	0.06	1.8 (-1.4 to 5.0)	0.26
6 months	70	-0.7 (-3.3 to 2.0)	100	-1.7 (-4.0 to 0.5)	1.8 (-1.6 to 5.2)	0.30	1.1 (-2.4 to 4.6)	0.55
Change in lean body mass (kg)								
6 months	71	-0.2 (-0.6 to 0.2)	100	-0.1 (-0.5 to 0.2)	-0.0 (-0.6 to 0.5)	0.88	-0.1 (-0.7 to 0.4)	0.63
Change in fat mass (kg)								
6 months	71	-0.3 (-0.9 to 0.4)	100	0.0 (-0.4 to 0.4)	-0.2 (-0.8 to 0.5)	0.65	-0.3 (-0.9 to 0.4)	0.37
Change in whole-body bone mineral content (g)								
6 months	71	14.7 (-2.2 to 31.6)	100	4.3 (-9.9 to 18.6)	8.3 (-14.8 to 31.4)	0.48	10.4 (-11.7 to 32.5)	0.36
Change in whole-body BMD (g/cm^2)								
6 months	71	0.005 (-0.001 to 0.010)	100	0.001 (-0.003 to 0.006)	0.001 (-0.007 to 0.009)	0.79	0.003 (-0.004 to 0.011)	0.39
Femoral neck BMD (g/cm^2)								
6 months	71	0.011 (0.004 to 0.018)	100	0.003 (-0.003 to 0.009)	0.008 (-0.002 to 0.017)	0.10	0.008 (-0.001 to 0.017)	0.08
Change in total hip BMD (g/cm^2)								
6 months	71	0.001 (-0.008 to 0.011)	100	0.006 (-0.002 to 0.013)	-0.007 (-0.019 to 0.005)	0.25	-0.004 (-0.016 to 0.008)	0.50
Change in lumbar spine L1-L4 BMD (g/cm^2)								
6 months	71	0.014 (0.000 to 0.029)	100	0.011 (-0.001 to 0.023)	-0.001 (-0.021 to 0.018)	0.89	0.003 (-0.015 to 0.022)	0.72
Change in weekly physical activity (MET minutes)								
12 weeks	74	1710 (328 to 3092)	89	638 (-797 to 2073)	2269 (-144 to 4683)	0.07	1072 (-920 to 3064)	0.29
6 months	63	394 (-1151 to 1938)	87	928 (-386 to 2242)	98 (-2021 to 2216)	0.93	-534 (-2562 to 1494)	0.60
Change in general physical health (SF-12)								
12 weeks	82	-1.3 (-2.5 to -0.0)	104	-1.7 (-2.8 to -0.6)	0.5 (-1.3 to 2.3)	0.59	0.4 (-1.3 o 2.1)	0.64
6 months	70	-2.0 (-3.4 to -0.6)	100	-1.3 (-2.5 to -0.2)	-0.5 (-2.4 to 1.3)	0.57	-0.7 (-2.4 to 1.1)	0.46
Change in general mental health (SF-12)								
12 weeks	82	-0.1 (-1.6 to 1.4)	104	-0.8 (-2.2 to 0.5)	0.8 (-1.3 to 2.9)	0.49	0.8 (-1.3 to 2.8)	0.46
6 months	70	1.1 (-0.6 to 2.9)	100	-1.5 (-2.9 to 0.0)	2.5 (0.2 to 4.8)	0.03	2.6 (0.3 to 4.9)	0.03

* Risk variables used to adjust for confounding when analysing adherence: smoking, alcohol consumption, employment status, education, marital status, disease stage, Gleason score, treatment, and co-morbidities

ESM Table 5 Safety outcomes for PP population

	Football group (n=82)	Usual care group (n=105)
Safety outcomes		
Falls	9	6
p-value p=0.17		
Fractures	1	2
p-value p=1.00		
Serious adverse events(admissions)		
One	7	8
Two	0	7
Total	7	22
p-value p=0.07		

ESM Table 6 Injuries in football group

	Number of injuries	Percent of all injuries
Reason for injury		
All injuries	60	100
Overuse injuries	19	32
Acute/traumatic injuries	41	68
Injured region		
Chest/ribs/upper back	4	7
Lower back/sacrum/Ilium	2	3
Shoulder/clavicle	1	2
Wrist	1	2
Hand/fingers	1	2
Hip/groin	3	5
Thigh	18	30
Knee	5	8
Lower leg/Achilles tendon	21	35
Ankle	4	7
Injury type		
Bent rib cage	1	2
Dislocation/subluxation	2	3
Sprains	11	18
Lesion of meniscus/cartilage	1	2
Tendon irritation	10	17
Partial tendon rupture	1	2
Tendon rupture	1	2
Hematoma/contusion	2	3
Excoriation/scratch	1	2
Muscle strain	29	48
Bursa irritation	1	2

ESM Figure 1. Weekly physical activity, according to allocation group

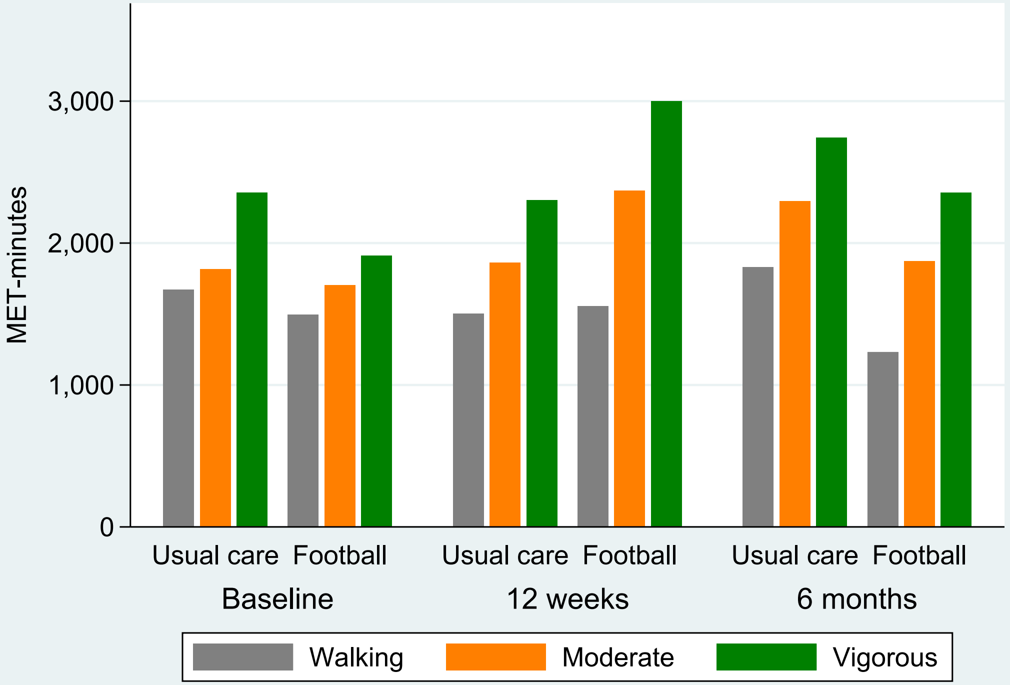

Supplementary centre level information

Scanners used for Dual-energy X-ray Absorptiometry: Aarhus: Discovery A, Hologic; Copenhagen: Lunar Prodigy Advanced Scanner, GE Healthcare; Esbjerg: Discovery A, Hologic; Odense: Discovery A, Hologic.

Participants enrolled and randomised at each centre: Copenhagen (n=106), Aarhus (n=56), Esbjerg (n=37) and Odense (n=15).
